# Genetic Estimates for Growth and Shape-Related Traits in the Flatfish Senegalese Sole

**DOI:** 10.3390/ani11051206

**Published:** 2021-04-22

**Authors:** Israel Guerrero-Cozar, Eduardo Jimenez-Fernandez, Concha Berbel, Elena Espinosa, Manuel Gonzalo Claros, Ricardo Zerolo, Manuel Manchado

**Affiliations:** 1 IFAPA Centro El Toruño, Junta de Andalucía, Camino Tiro Pichón s/n, 11500 El Puerto de Santa María Cádiz, Spain; israel.guerrero@juntadeandalucia.es (I.G.-C.); mariac.berbel@juntadeandalucia.es (C.B.); 2 Free Radical Research Group, Centre for Health Sciences, University of the Highlands and Islands, Inverness IV2 3JH, UK; eduardojf85@icloud.com; 3 Molecular Biology and Biochemistry Department, University of Málaga, 29071 Málaga, Spain; elenamariaesga@gmail.com (E.E.); claros@uma.es (M.G.C.); 4 CIBER de Enfermedades Raras (CIBERER), 29071 Málaga, Spain; 5 Institute of Biomedical Research in Málaga (IBIMA), IBIMA-RARE, 29010 Málaga, Spain; 6 Instituto de Hortofruticultura Subtropical y Mediterránea (IHSM-UMA-CSIC), 29010 Málaga, Spain; 7 CUPIMAR, Ctra. Carraca, n° 2 Salina San Juan Bautista, San Fernando, 11100 Cádiz, Spain; ricardo.zerolo@cupimar.com; 8 “Crecimiento Azul”, Centro IFAPA El Toruño, Unidad Asociada al CSIC, 11500 El Puerto de Sta María, Spain

**Keywords:** Senegalese sole, genetic estimates, shape, growth, breeding

## Abstract

**Simple Summary:**

To increase competitiveness, the aquaculture flatfish industry demands animals with optimal growth rates and a high shape quality. Genetic breeding is an essential tool to achieve these goals but it requires the estimation of the genetic components of these traits under industrial conditions. The current study provides phenotypic data and genetic parameters of eight traits related to growth and shape quality. The high heritabilities and correlations obtained support that genetic breeding programs can be successfully implemented in Senegalese sole to optimize production.

**Abstract:**

Shape quality is very important in flatfish aquaculture due to the impact on commercialization. The Senegalese sole (*Solea senegalensis*) is a valuable flatfish with a highly elliptic body that slightly changes with age and size, and it is prone to accumulating malformations during the production cycle. The present study aims to investigate the genetic parameters of two growth traits (weight and standard length) and six shape quality predictors (ellipticity, three body heights (body height at the pectoral fin base [BHP], body maximum height [BMH] and caudal peduncle height [CPH]) and two ratios (BMH/BHP and BMH/CPH)). These traits were measured before the on-growing stage (age ~400 days (d)) and at harvest (~800 d). Phenotypic data, heritabilities and genetic and phenotypic correlations between the traits are presented and discussed. High or very high heritabilities (0.433–0.774) were found for growth traits, body heights and ellipticity and they were higher at 400 than 800 d. In contrast, the ratios of BMH/BHP and BMH/CPH were less heritable (0.144–0.306). Positive and very high (>0.95) correlations between growth traits and the three heights were found and decreased with age. In contrast, ellipticity had negative and medium-high genetic correlations with growth traits and heights, indicating fish selected for bigger size would also become rounder. The ratio of BMH/CPH showed low genetic correlations with all traits and provided complementary information to ellipticity for a better fitting to the expected lanceolate body morphology of sole. The genetic correlations for all traits at both ages were very high, indicating that selection before entering the growth-out stage in recirculation aquaculture systems is recommended to accelerate genetic gains.

## 1. Introduction

Flatfish is a general name for a diverse group of highly appreciated species worldwide, both in fisheries and aquaculture. They are morphologically unique among fishes due to their body asymmetry, which is acquired after the migration of one eye to the opposite side and the cranium remodeling in early larval stages. This process, known as metamorphosis, also entails a drastic reorganization of the abdominal cavity, skin pigmentation patterns and the development of sensory structures for the adaptation to a bottom-dwelling mode of life. As a consequence, the new flattened bodies acquire species-specific shapes for swimming, and camouflage capabilities as adaptive mechanisms to specific ecological niches [1]. In a general way, flatfish species from the families Bothidae, Cynoglossidae, Poecilopsettidae and Soleidae are characterized by oblong bodies with shorter jaws and longer dorsal and anal fins than the families Citharidae, Paralichthyidae, Pleuronectidae or Scophthalmidae, among others [2]. Although flatfish shape is slightly modified with the age and size, the species-specific morphological features are well-identified by consumers and are usually important criteria in commercial decisions and the price of fresh marketed products. Due to the high relevance of external morphology on commercialization, the production of high-quality shaped fish is highly important in aquaculture to enhance consumers’ awareness and support their perception of fish aquaculture products [3].

Senegalese sole (*Solea senegalensis*) is a marine flatfish of high economic value whose aquaculture is rapidly growing in Southern Europe. The shape of this right-eyedflatfish is well-recognized by the lanceolate bodies, short jaws and long fins. However, this species exhibits a high plasticity of the skeletal components, such as the vertebral number, which oscillates between 44 and 48 (mode = 45) with 8–9 in the abdominal region, 34–35 in the caudal region and 3–4 in the caudal complex [4,5]. Moreover, this species is highly prone to vertebral abnormalities and other skeletal malformations that can reach even more than 70% of individuals in cultured populations, most of them corresponding to vertebral fusions in the caudal region and deformities in the caudal complex [4,6,7,8,9]. Most of these malformations are usually externally unnoticed or they have a moderate effect on gross phenotypic morphology (approximately 46% of animals with vertebral deformities were categorized as normal) [4]. However, this plasticity and high incidence of malformations can shift the body ellipticity with an impact on the quality of the marketable product; hence, it is very important to identify the phenotypic and genetic determination of the main morphological traits and the association with other productive parameters.

Nutritional factors and environmental conditions have been identified as two major modulators of morphological features and malformations in Senegalese sole. High levels of vitamin A increase the mean number of vertebrae and the malformation rates in the vertebrae and caudal fin [10]. Moreover, a high stocking density (29.8 kg m^−2^) shifts the relative body proportions toward a wider head and a shortened caudal region with an enlarged peduncle [11]. A high temperature (>18 °C) during larval rearing also increased vertebral anomalies in the caudal region and caudal complex, although the effects on external morphology were not evaluated [7]. In the closely related species *Solea solea*, the body ellipticity measured using image analysis was proposed as an optimal trait to assess the quality of external sole shape [12]. This trait showed a moderate heritability (0.34 ± 0.11) and a moderate and negative genetic correlation with body weight, highlighting the importance of controlling for this trait to maintain high-quality shaped fish in genetic breeding programs [12]. This study aimed at estimating the genetic and phenotypic parameters for growth and shape-related traits at two important stages in the production cycle of Senegalese sole, before entering growth-out in recirculation aquaculture systems (RAS) (~400 days (d)) and at harvest (~800 d). Weight, standard length, three body heights (at the pectoral fin, maximal and in the peduncle), their relative ratios and body ellipticity were evaluated as quality indicators of sole shape. Heritability estimates and genetic and phenotypic correlations at both ages are provided. The data provided are highly relevant in genetic breeding programs.

## 2. Materials and Methods

### 2.1. Animals

Broodstock used to produce families comprised 150 wild specimens approx. 8 years old caught in salt marshes from the Gulf of Cadiz (Spain). They were fed with frozen feed including mussels, small squids and polychaeta worms (Seabait Ltd., Ashington, UK) on alternative days. Mass spawning strategy to create the families was previously described [13]. Briefly, spawning was synchronized by thermoperiod control [14]. Due to the courtship behavior of sole [15], it is not easy to achieve all the breeder tanks (*n* = 9) responding simultaneously in the same thermocycle. Hence, with the objective to increase the number of families in the population upon evaluation, seven evaluation batches (EBs) obtained after different thermocycles were created by mixing proportionally the volume of eggs from each tank that contributed offspring in each thermal treatment. To facilitate the data comparison and convergence, the offspring of a breeder tank (*n* = 6) were always included in all EBs. Larval rearing and weaning protocols for each EB were those previously described [16,17] and each EB was always managed as a unit until harvest without any grading.

**Figure 1:**
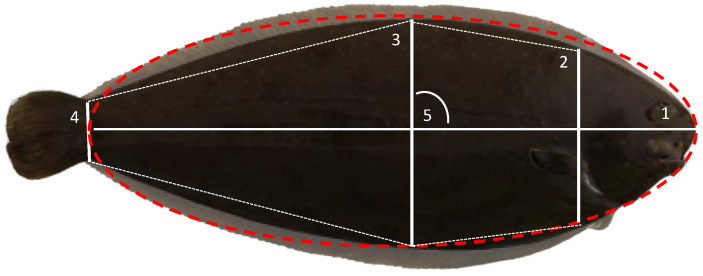
Shape measurements: 1: standard length (SL); 2: body height at the insertion of the pectoral fin (BHP); 3: body maximum height (BMH); 4: caudal peduncle height (CPH); and 5: ellipticity ((SL − BMH)/(SL + BMH)). A theoretical ellipse fitting the horizontal axis from the mouth tip to the peduncle center and the vertical axis to BMH is indicated by red dashed line.

For genetic evaluation, fish (ranging from 200 to 550 specimens per EB) were intraperitoneally tagged, with ages ranging between 150 and 278 days post-hatch (dph) as previously reported [13,18]. Later, fish were phenotypically evaluated in vivo at ~400 d (ranging from 395 to 446 dph) before entering the growth-out period in RAS and at harvest age ~800 d (ranging between 733 and 861 dph). No intermediate samplings were carried out to follow standard production practices and minimize animal handling and stress. Cumulative mortality between ages was lower than 5% and a total of 1840 fish (EB1 = 136; EB2 = 289; EB3 = 273; EB4 = 420; EB5 = 229; EB6 = 234; EB7 = 259) sampled at both ages were considered in this study. Information about the full dataset and culture conditions was previously reported [13]. Fish were individually weighted (W) using Gram FC-200 and a photograph was taken using a Canon EOs1300D camera following the methodology previously established in PROGENSA^®^ [19]. Image analysis was carried out using the Fiji 2.0.0-rc-69/1.52p and standard length (SL), body height at the insertion of the pectoral fin (BHP), body maximum height (BMH) and caudal peduncle height (CPH) were measured (Figure 1). The two ratios between heights (BMH to BHP and BMH to CPH) and ellipticity ((SL-BMH)/(SL + BMH)) [12] were calculated. At harvest, fish were sacrificed using slurring ice following commercial techniques and 60 specimens of each batch were kept alive as future breeders. From sacrificed fish were taken a piece of caudal fin that was preserved in 99% alcohol, and alive fish were sampled for blood by puncturing in the caudal vein using a heparinized syringe, adding heparin (100 mU) and keeping at −20 °C until use. All fish were sexed and the presence of white nodules compatible with amoebic disease (AD) were recorded.

### 2.2. DNA Isolation and Parentage Assignment

DNA isolation from blood (broodstock and non-sacrificed offspring) or caudal fin (slaughtered F1; 30 mg) was carried out using the Isolate II genomic DNA kit (Bioline, London, UK) following the manufacturer’s instructions. DNA was quantified using a Nanodrop ND-8000 and quality was evaluated by agarose gene electrophoresis. Genotyping of breeders and offspring was carried out using an 11-loci supermultiplex PCR [20] on an ABI3130 sequencer (Applied Biosystems, Foster City, CA, USA) and genotypes were collected using Genemapperv3.8 (Applied Biosystems, Foster City, CA, USA). Finally, parentage assignment was performed with Vitassign v8.2.1 [21] following the allelic exclusion method. Assignment rates to a single parent pair was 100%. A total number of 71 families from 37 males and 30 females were evaluated. The number of families per batch ranged from 11 (EB1 and EB5) to 23 (EB7). Offspring of seven males and six females were represented in four or more EB.

### 2.3. Statistical Analysis and Genetic Parameters

All data were tested for normality and homogeneity of variance using SPSS v.23 (SPSS, Chicago, IL, USA). Weight at 400 and 800 d were cube square root and square root transformed, respectively, to fit normality. ANOVA analysis using the General Linear. Models (GLM) procedure was carried out using the gender, EB and AD as fixed factors. To test the effect of age (evaluation of traits between 400 and 800 d), a repeated measures ANOVA was carried out for each trait using the same fixed factors. Regression analysis and slope significance testing were carried out with Prism 9.0 (Graphpad Software Inc., San Diego, CA, USA). Genetic estimates of heritability and correlations were calculated using restricted maximum likelihood adjusted linear mixed models (REML) in WOMBAT [22]: y = Xβ + Zu + e, where y is the observed trait, β is the fixed factor vector (gender, EB and AD), u is the animal random factor vector and e is the error.

## 3. Results

### 3.1. Phenotypic Data for Growth Traits

**Figure 2:**
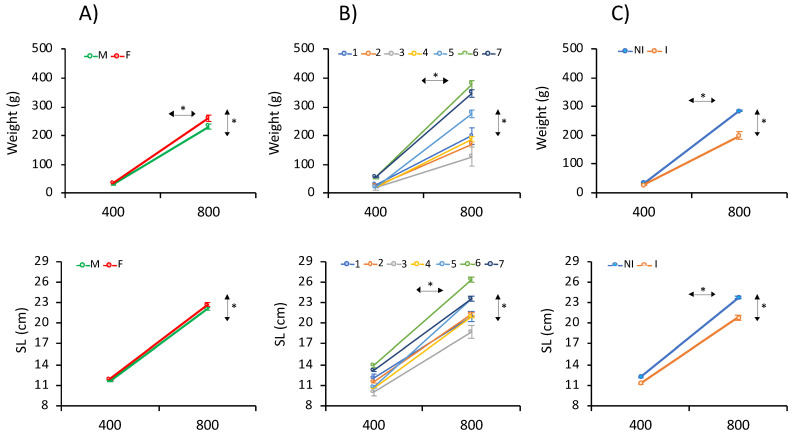
Estimated marginal means for weight and standard length (SL) as determined by repeated-measures ANOVA at 400 and 800 days (d) for (**A**) gender (male: M; female: F), (**B**) Evaluation Batch (EB) (1–7) and (**C**) Amoebic disease (AD) (infected: I; non-infected: NI). The asterisks (*) on the horizontal or vertical arrows denote if within- or between-subject effects were significant.

The phenotypic mean ± Standard error (SE) of growth traits (weight and SL) at 400 and 800 d are depicted in Table 1 and in Figure S1. Mean weight and SL were 32.4 ± 29.2 g and 12.00 ± 2.87 cm at 400 d and 264.9 ± 171.9 g and 23.35 ± 4.79 cm at 800 d. Statistical ANOVA analysis showed statistically significant differences associated with the gender, EB and AD (Figure 2 and Figure S1) for both traits. Estimated marginal means indicated that the females appeared on average 16.1% heavier and 2.8% longer than males at 400 d, and 12.2% heavier and 2.5% longer than males at 800 d (Figure 2 and Figure S1). A significant gender × EB interaction was observed at both ages. In addition, significant differences associated with the EB (*p* < 0.05) were found that ranged between 18.9 and 63.3 g at 400 d for EB3 and EB7, respectively, and between 126.8 and 376.7 g at 800 d for EB3 and EB6, respectively (Figure 2 and Figure S1). A total of 15.3% of evaluated fish at harvest had nodules compatible with amoebic lesions in the gut and/or liver. Fish without hepatic or intestinal amoebic lesions at 800 d were significantly heavier (44.9%) than infected fish (Figure 2). A repeated measures ANOVA analysis revealed significant age × EB and age × AD interactions for weight and length gain and age × gender for weight gain. Tendencies for the three different fixed factors and levels are depicted in Figure 2. A regression weight-length analysis for gender at both ages showed that the coefficients of determination (R^2^) were ≥ 0.95 with slopes between 3.32 and 3.34 (not statistically significant) (Figure S2).

### 3.2. Phenotypic Data for Height Traits

**Figure 3:**
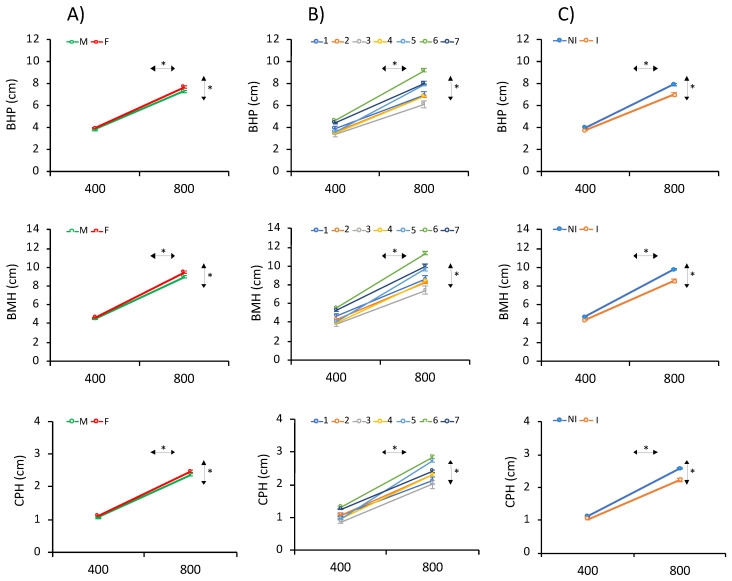
Estimated marginal means for height at the pectoral fin base (body height at the insertion of the pectoral fin (BHP), body maximum height (BMH) and caudal peduncle height (CPH) as determined by repeated-measures ANOVA at 400 and 800 days (d) for (**A**) gender (male: M; female: F), (**B**) Evaluation Batch (EB) (1–7) and (**C**) Amoebic disease (AD) (infected: I; non-infected: NI). The asterisks (*) on the horizontal or vertical arrows denote if within- or between-subject effects were significant.

Due to the flattened morphology of sole, the body height at the insertion of the pectoral fin (BHP), body maximum height (BMH) and caudal peduncle height (CPH) and the two ratios of BMH/BHP and BMH/CPH were determined both at 400 and 800 d (Table 1; Figure 1). Mean BHPs were 3.91 ± 1.01 and 7.74 ± 1.77 cm at 400 and 800 d, respectively; the BMHs were 4.62 ± 1.34 and 9.53 ± 2.35 cm, respectively; and the CPHs were 1.11 ± 0.34 and 2.61 ± 0.67 cm, respectively. The three height traits showed statistically significant differences associated with the gender and EB at both ages, and AD at 800 d (Figure 3). Females and non-infected soles had higher heights than males and infected fish. On average, heights in females were 4.6, 4.5 and 4.3% higher than in males and the non-infected fish, and 10.2, 11.2 and 11.9% higher than in infected fish, for BHP, BMH and CPH, respectively. Moreover, EB6 and EB3 showed the largest and lowest heights, respectively. A longitudinal analysis to determine the height gain from 400 to 800 d using repeated measures ANOVA demonstrated significant interactions of age × gender, age × EB and age × AD (Figure 3).

A regression analysis of CPH and BHP on BMH indicated a stronger association between BHP and BMH (R^2^ > 0.97) than CPH and BMH (R^2^ > 0.86). Moreover, slopes for males were statistically significantly smaller than females at 800 d at both ages (Figure S3).

**Figure 4:**
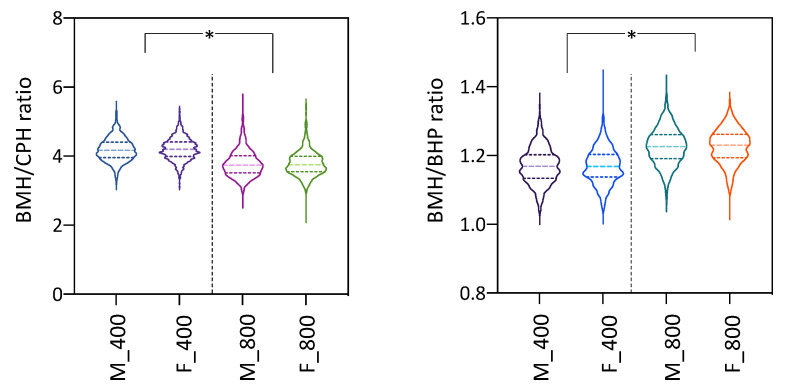
Violin plots for body maximum height (BMH) to caudal peduncle height (CPH) and BMH to body height at the insertion of the pectoral fin (BHP) ratios. Data for males (M) and females (F) at both 400 and 800 days (d) are indicated. The asterisk (*) denotes statistically significant differences between ages.

With respect to the BMH/CPH and BMH/BHP ratios, BMH/CPH significantly reduced and BMH/BHP increased with age, from 400 to 800 d (Figure 4). A significant effect of the EB on both ratios at 400 and 800 d was detected (Figure S4). Nevertheless, the gender effect was only significant for BMH/BHP at 400 d. The longitudinal analysis only identified a significant interaction of age × EB (Figure S4). In the repeated-measures ANOVA a significant between-subject effect of AD for BMH/BHP was also found.

### 3.3. Phenotypic Data for Ellipticity

**Figure 5:**
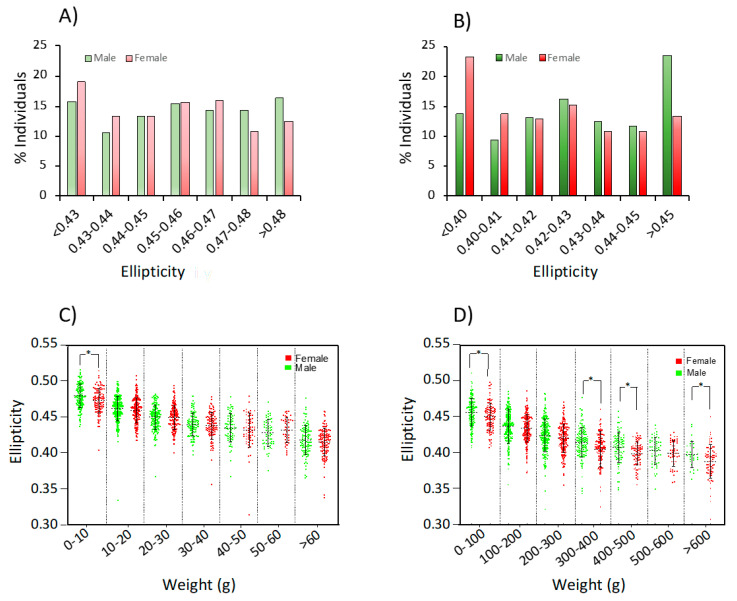
Distribution of ellipticity classes by weight. Panels (**A**) (400 days (d)) and (**B**) (800 d) show the frequency of males (green) and females (red) by ellipticity classes. Panels (**C**) (400 d) and (**D**) (800 d) show the ellipticity scatterplot by weight class and gender. The asterisks (*) denote statistically significant differences between gender in a weight class.

**Figure 6:**
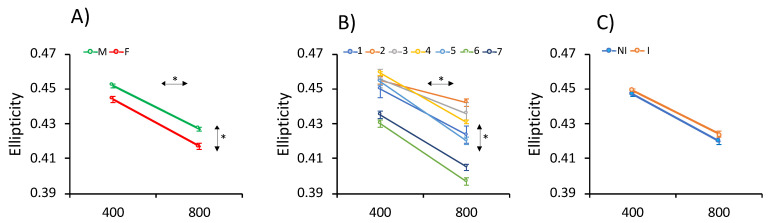
Estimated marginal means for ellipticity as determined by repeated-measures ANOVA at 400 and 800 days (d) for the (**A**) gender (male: M; female: F), (**B**) Evaluation Batch (EB) (1–7) and (**C**) Amoebic disease (AD) (infected: I; non-infected: NI). The asterisks (*) on the horizontal or vertical arrows denote if within- or between-subject effects were significant.

Mean ellipticity was 0.449 ± 0.025 at 400 d and 0.422 ± 0.029 at 800 d (Table 1). The distribution of ellipticity at both shapes is shown in Figure 5. Values ranged from 0.32 to 0.52 at 400 d and between 0.24 and 0.51 at 800 d. ANOVA analysis indicated statistically significant differences associated with the gender and EB at both ages and with AD at 800 d (Figure 6). Males and infected fish were more elliptic than females (1.0 and 2.3% higher at 400 and 800 d, respectively) and non-infected fish (1.4% higher) (Table 1; Figure 6). The longitudinal analysis demonstrated a significant interaction of age × gender and age × EB during the cultivation period in RAS (Figure 6).

As ellipticity was significantly and negatively correlated with weight (R^2^ ranging from 0.362 to 0.443), an ANCOVA analysis using the weight as a covariate was carried out and significant differences associated with the EB and gender at both ages were still observable. An analysis of ellipticity by weight class indicated that females were statistically less elliptic than males in class 0–10 g at 400 d and classes 0–100, 300–400, 400–500 and > 600 g at 800 d.

### 3.4. Genetic Estimates

#### 3.4.1. Heritability

Heritabilities and correlations for growth and shape-related traits at 400 and 800 d are depicted in Table 2. Heritabilities were higher for all the traits (except BMH/CPH) at 400 than 800 d. Heritability estimates for weight, SL, the three heights and ellipticity were high or very high at both ages. They ranged between 0.567 and 0.774 at 400 d and between 0.433 and 0.735 at 800 d for ellipticity and SL, respectively. The ratios of BMH/BHP and BMH/CPH had low or moderate heritability values (0.270–0.303 at 400 d and 0.144–0.306 at 800 d).

#### 3.4.2. Genetic Correlations

Genetic correlations between growth and height traits were very high both at 400 and 800 d (>0.95). The ratio of BMH/BHP had moderate-high genetic correlations with growth and height traits that were higher at 400 (0.858–0.881) than 800 d (0.412–0.612). The genetic correlations of BMH/CPH were low (<0.28). The ellipticity had negative and high genetic correlations with growth and height traits ranging from −0.724 to −0.828 at 400 d and from −0.509 to −0.733 at 800 d, and a negative and low correlation with height ratios (Table 2).

Genetic and phenotypic correlations between both ages are depicted in Table 3. Ellipticity (0.912) had the highest genetic correlation when the same traits were compared at 400 and 800 d, followed by growth and height traits (average 0.825 and 0.874). The lowest values were between height ratios (0.663–0.687).

## 4. Discussion

Genetic breeding programs for growth performance and shape quality are essential for flatfish aquaculture industry competitiveness. This highly plastic taxonomic group transforms during development from a bilateral symmetry to an asymmetric, highly specialized flattened body. Evolutionary studies have demonstrated that different ecological traits act as a driver of body shape in flatfish, acquiring a wide range of body depths, jaw lengths and fin lengths [2]. Hence, flatfish families can be identified by specific shapes and morphological features that should be carefully preserved in aquaculture to maintain consumer acceptance and commercial value. In the case of Senegalese sole, body shape is expected to be highly elliptic and lanceolate with short jaws and long dorsal and anal fins that contrast with the shape of most pleuronectids or scophthalmids with deeper bodies, longer jaws and short dorsal and anal fins. However, several reports that dealt with morphological traits in Senegalese sole in aquaculture reported high rates of malformations that in most cases do not have a severe impact on external gross morphology [4,6,7,8]. In this study, we investigate for first time in Senegalese sole the phenotypic and genetic variation associated with shape-related traits under industrial conditions in RAS. These results are highly valuable to design genetic breeding programs and integrate the shape quality within the selection schemes.

The reproduction of Senegalese sole is extremely complex due to three singularities: the courtship behavior, the dominance and fidelity of highly successful spawners and the low production of sperm [15,23]. Taking into account these reproductive limitations, this study produced families by mass-spawning using a wild broodstock distributed in nine tanks after spawning synchronization by thermoperiod control [14,23]. Although tagging, stocking density, water temperature, type of tank or feed were common to all EBs, this factor had an important effect on growth and shape-related traits after the RAS growth-out phase. The longitudinal analysis (since the same subset of tagged soles was analyzed at both ages) showed different tendencies in RAS even between EBs with a very similar genetic structure and age at sampling, suggesting that some additional factors such as social interactions or differences in the actual flow-through dynamics could also play a key role in the evaluated traits.

In addition to the EB, gender also had an important effect on growth and shape-related traits. The females appeared 12.2–16.1% heavier and 2.1–2.8% longer than males in the evaluation period. These data are in agreement with the differences previously found in sole juveniles and at harvest [13,18,24]. Moreover, in this study we demonstrate that females are less elliptic than males even after correcting by weight. These differences were more evident at harvest, probably due the ovary maturation increasing the abdominal cavity, which in turn reduces ellipticity. A regression analysis between heights also evidenced a small change in the slopes by gender that was not clearly observable when height ratios were analyzed, indicating that compensatory mechanisms could modify the relative body proportions. In addition to gender effects, the presence of amoebic nodules in liver or intestine at harvest also influenced growth and shape-related traits. This parasite accumulates mainly in the intestinal mucosa and later spreads to some different tissues [25]. Although mortality is scarce, this study demonstrated that non-infected fish were 44.9% heavier than infected fish. Moreover, the infected fish were slightly less elliptic at harvest even after correcting by weight, and changed the ratio of BMH/BHP due to the excess of nodules that in some cases distorted the size of the abdominal cavity.

The ellipticity of the sagittal plan was proposed as the best trait to measure shape quality in sole since this trait could be easily derived from direct measures on fish (using body height and body length) or by image analysis, fitting theoretical ellipses with a similar precision [12]. This theoretical assumption is based on the expected elongated body shape of soleids that differentiates them from pleuronectids or scophthalmids. Although this trait is highly influenced by body size, and bigger fish tend to be rounder, the ellipse fitting still remains as a good predictor of shape for soles. Our ellipticity data confirmed a major effect of weight on ellipticity distribution, with bigger values at 400 than 800 d and a progressive reduction with bigger weight class sizes (Figure 5). Moreover, as indicated above, females were rounder than males even after correcting by weight due to the increase in abdominal size for sexual maturation. In yellowtail flounder, females had relatively deeper abdomens and larger heads than males [26]. However, these differences associated with gender were not observed in *S. solea*, although these authors did not follow a longitudinal approach or provide information about gonad development that could explain such differences. The significant effects of EB conditions, as indicated above, on the ellipticity trajectory also denote the importance of culture conditions on shape, and the relevance of controlling this important feature to maintain high shape-quality standards for fish commercialization.

Heritabilities for growth traits in flatfish are highly influenced by the age and production system. Previous studies in Japanese flounder reported high or very high heritabilities (>0.6) for growth traits (weight and body length) in juvenile stages (<300 dph) that gradually decreased in older flatfish [27,28,29,30]. In contrast, heritabilities for weight at harvest in the closely related species *S. solea*, cultivated in RAS, were low-moderate (0.23–0.25), although this species has very low growth rates compared to *S. senegalensis* [12,31,32]. A previous study in Senegalese sole with a higher number of fish (*n* = 2171 offspring) also estimated higher heritabilities for growth traits in juveniles before growth-out than later at harvest [13]. These results indicate that the high densities reached in RAS, the water flow dynamics and social hierarchies could mask genetic effects in spite of this species still maintaining high growth rates [33,34]. RAS is not a natural environment for sole, modulating the genetic effects on growth, as determined in common sole with a clear genotype, by environment interaction [35]. Moreover, most females at harvest showed some degree of gonad maturation that was reported in rainbow trout to have a huge impact on the additive genetic variability of weight [36]. Interestingly, the high genetic correlation between 400 and 800 d for growth traits support that growth parameters estimated in juvenile stages before RAS could be used as a good predictor of growth performance later at harvest.

Shape predictors such as ellipticity had very high heritabilities (>0.74) at both ages. These values were considerably higher than those obtained in *S. solea* (0.34) [12] or *Oreochromis niloticus* (0.12–0.45) [37,38]. Although some nutritional, management and culture conditions were reported as regulators of meristic characters and malformation rates in sole [7,10,11], our results indicate a high additive genetic component of the external shape-related traits evaluated in this study. Although malformations could exist (they were not evaluated in this study), most of them would have a low impact on gross morphology as previously indicated [4], evidencing a high genetic component for ellipticity. Nevertheless, further studies are required to associate the shape traits with the skeletal characteristics in order to understand the main causes behind the ellipticity range. Moreover, the high genetic correlations (0.91) between both ages confirm that those genetic factors controlling shape are already acting in juveniles, and hence selection could also be carried out in juveniles.

It should be noted that ellipticity was dependent on fish size. In *S. solea*, a moderate negative genetic correlation (r_g_ = −0.44) between ellipticity and weight was reported [12]. Similarly, in this study, a high and negative genetic correlation between both traits at 400 (−0.768) and 800 d (−0.608) was determined, indicating that fish reaching a bigger size were also rounder. These negative correlations should be carefully considered if selection for increased weight at harvest is carried out, since less elliptic fish will be produced. Since most soles are sold in fresh markets, ellipticity was proposed as a correction factor for weight-targeted selection breeding programs [12]. A combined selection index setting a zero change in shape reduced by 9.9–13.8% the response to harvest weight that is assumed to preserve a high-quality shape standard [12]. However, no correction would be necessary if, finally, the industry moves toward transformed seafood products, which is one of the most promising markets for flatfish.

The three heights showed a positive and very high genetic correlation with growth traits (>0.95). However, the heritabilities for the two height ratios were low-moderate and the BMH/CPH ratio had very low genetic correlations with growth and height traits. This latter ratio is strongly related to the swimming speed and performance [39,40]. A deep caudal peduncle provides the fish with a superior ability to accelerate and greater power for propulsion, allowing it to reach a high swimming speed and efficiency [39]. Soles are usually very sedentary in the tanks and they do not require high water columns since they are passive feeders in the tank bottom. Hence, there is not expected to exist a high selection pressure on swimming efficiency in the RAS, although fish should adapt to water currents in the tanks. However, this ratio seems to be useful to refine a lanceolate shape toward a more theoretical elliptic one that fits better to the sole body structure (Figure S5). The peduncle is usually considered as the caudal reference point for body length since the caudal fin is highly variable in size and morphology and the BMH/CPH ratio increases with age (Figure 4). Highly pronounced ratios (due to higher BMH or lower CPH) are associated with very high lanceolate shapes (Figure S5) that deviate from the symmetrical body ellipse, giving rise to turbot-like morphologies. The low heritability for this trait could be due to the benthic way of life and the sensitivity of the caudal complex to traumatisms and malformations that in turn can remodel the peduncle. The low genetic correlations with other ellipticity and growth traits indicate that this trait can provide new relevant information for a genetic selection index to preserve a sole high-quality shape.

## 5. Conclusions

This study provides phenotypic and genetic estimates for growth and shape-related traits and supports selective breeding as an effective strategy to improve these traits in Senegalese sole. The gender, EB and AD had significant effects on most traits when evaluated using a longitudinal approach, indicating that these factors need to be carefully controlled to achieve accurate estimates. Moreover, the high correlations at both ages support that selection can be carried out before growth-out in RAS, accelerating the breeding cycles. A combination of ellipticity, BMH/CPH and weight could be used in a multi trait selection index to control the roundness associated with weight gain and select animals with an optimal lanceolate morphology and growth rate.

## Figures and Tables

**Table 1:** Phenotypic data for growth traits (weight and standard length (SL)), heights (body height at the insertion of the pectoral fin (BHP), body maximum height (BMH) and caudal peduncle height (CPH)), height ratios (BMH/BHP and BMH/CPH) and ellipticity at 400 and 800 days (d). Overall mean ± standard error by gender is shown. The number (*n*) of soles evaluated at each age is also indicated.

400 days (*n* = 1840)	Male (*n* = 1007)	Female (*n* = 833)	Mean
Weight	30.7 ± 28.0	34.4 ± 30.4	32.4 ± 29.2
SL	11.85 ± 2.87	12.18 ± 2.86	12.00 ± 2.87
BHP	3.84 ± 1.00	4.00 ± 1.02	3.91 ± 1.01
BMH	4.54 ± 1.33	4.71 ± 1.34	4.62 ± 1.34
CPH	1.13 ± 0.34	1.10 ± 0.34	1.11 ± 0.34
BMH/BHP	0.45 ± 0.03	0.45 ± 0.03	0.45 ± 0.03
BMH/CPH	1.17 ± 0.06	1.17 ± 0.06	1.17 ± 0.05
Ellipticity	4.165 ± 0.358	4.183 ± 0.335	4.173 ± 0.347
**800 days (*n* = 1840)**	**Male (*n* = 1007)**	**Female (*n* = 833)**	**Mean**
Weight	244.0 ± 153.0	290.3 ± 189.3	264.9 ± 171.9
SL	22.91 ± 4.64	23.88 ± 4.91	23.35 ± 4.79
BHP	7.51 ± 1.66	8.01 ± 1.85	7.74 ± 1.77
BMH	9.24 ± 2.22	9.87 ± 2.46	9.53 ± 2.35
CPH	2.55 ± 0.66	2.68 ± 0.67	2.61 ± 0.67
BMH/BHP	0.426 ± 0.027	0.419 ± 0.028	0.424 ± 0.028
BMH/CPH	1.225 ± 0.054	1.227 ± 0.050	1.226 ± 0.052
Ellipticity	3.650 ± 0.325	3.699 ± 0.320	3.673 ± 0.323

**Table 2:** Heritabilities (diagonal in bold), phenotypic correlations (below the diagonal) and genetic correlations (above the diagonal) for growth traits (weight (W) and standard length (SL)), heights (body height at the insertion of the pectoral fin (BHP), body maximum height (BMH) and caudal peduncle height (CPH)), height ratios (BMH/BHP and BMH/CPH) and ellipticity (E) at 400 days (d) (top) and 800 d (bottom).

400 d	W	SL	BHP	BMH	CPH	BMH/BHP	BMH/CPH	E
W	**0.625 ± 0.109**	0.991 ± 0.004	0.988 ± 0.004	0.992 ± 0.003	0.990 ± 0.005	0.874 ± 0.057	0.161 ± 0.189	−0.768 ± 0.073
SL	0.983 ± 0.002	**0.567 ± 0.104**	0.981 ± 0.007	0.986 ± 0.005	0.984 ± 0.007	0.881 ± 0.054	0.167 ± 0.189	−0.724 ± 0.085
BHP	0.981 ± 0.002	0.976 ± 0.002	**0.623 ± 0.110**	0.991 ± 0.001	0.948 ± 0.005	0.858 ± 0.064	0.284 ± 0.180	−0.738 ± 0.025
BMH	0.988 ± 0.001	0.982 ± 0.002	0.999 ± 0.001	**0.621 ± 0.109**	0.955 ± 0.004	0.878 ± 0.055	0.247 ± 0.183	−0.828 ± 0.056
CPH	0.952 ± 0.004	0.953 ± 0.004	0.974 ± 0.010	0.979 ± 0.008	**0.576 ± 0.105**	0.881 ± 0.055	0.044 ± 0.193	−0.749 ± 0.079
BMH/BHP	0.591 ± 0.024	0.61 ± 0.023	0.528 ± 0.028	0.622 ± 0.023	0.587 ± 0.024	**0.270 ± 0.069**	0.102 ± 0.035	−0.521 ± 0.034
BMH/CPH	0.062 ± 0.045	0.047 ± 0.043	0.09 ± 0.044	0.094 ± 0.044	−0.191 ± 0.044	0.076 ± 0.204	**0.303 ± 0.076**	−0.254 ± 0.045
E	−0.673 ± 0.031	−0.284 ± 0.033	−0.838 ± 0.054	−0.750 ± 0.025	−0.677 ± 0.031	−0.662 ± 0.115	−0.487 ± 0.153	**0.774 ± 0.117**
**800d**	**W**	**SL**	**BHP**	**BMH**	**CPH**	**BMH/BHP**	**BMH/CPH**	**E**
W	**0.486 ± 0.099**	0.983 ± 0.007	0.974 ± 0.01	0.978 ± 0.008	0.983 ± 0.008	0.546 ± 0.162	0.016 ± 0.198	−0.608 ± 0.115
SL	0.975 ± 0.002	**0.433 ± 0.094**	0.961 ± 0.015	0.957 ± 0.016	0.964 ± 0.014	0.412 ± 0.183	0.011 ± 0.198	−0.509 ± 0.137
BHP	0.967 ± 0.003	0.948 ± 0.005	**0.549 ± 0.105**	0.996 ± 0.002	0.953 ± 0.018	0.506 ± 0.177	0.181 ± 0.193	−0.703 ± 0.092
BMH	0.982 ± 0.002	0.962 ± 0.004	0.983 ± 0.001	**0.515 ± 0.102**	0.961 ± 0.016	0.586 ± 0.155	0.182 ± 0.192	−0.733 ± 0.085
CPH	0.928 ± 0.005	0.918 ± 0.006	0.911 ± 0.007	0.926 ± 0.006	**0.463 ± 0.097**	0.612 ± 0.151	−0.073 ± 0.195	−0.586 ± 0.123
BMH/BHP	0.479 ± 0.027	0.486 ± 0.027	0.345 ± 0.031	0.508 ± 0.026	0.474 ± 0.026	**0.144 ± 0.046**	0.178 ± 0.211	−0.389 ± 0.031
BMH/CPH	−0.003 ± 0.042	−0.015 ± 0.04	0.046 ± 0.043	0.050 ± 0.043	−0.314 ± 0.040	0.048 ± 0.029	**0.306 ± 0.075**	−0.217 ± 0.044
E	−0.557 ± 0.037	−0.447 ± 0.042	−0.644 ± 0.033	−0.662 ± 0.030	−0.548 ± 0.038	−0.719 ± 0.117	−0.534 ± 0.144	**0.735 ± 0.115**

**Table 3:** Genetic (top) and phenotypic correlations between 400 (left) and 800 days (d) (right) for growth traits (weight (W) and standard length (SL)), heights (body height at the insertion of the pectoral fin (BHP), body maximum height (BMH) and caudal peduncle height (CPH)), height ratios (BMH/BHP and BMH/CPH) and ellipticity (E).

	Genetic	800 d							
		W	SL	BHP	BMH	CPH	BMH/BHP	BMH/CPH	E
400 d	W	0.843 ± 0.054	0.831 ± 0.060	0.832 ± 0.057	0.838 ± 0.055	0.849 ± 0.054	0.509 ± 0.169	0.041 ± 0.194	−0.554 ± 0.122
	SL	0.828 ± 0.058	0.837 ± 0.057	0.813 ± 0.062	0.817 ± 0.061	0.826 ± 0.057	0.509 ± 0.161	0.049 ± 0.194	−0.511 ± 0.134
	BHP	0.868 ± 0.047	0.859 ± 0.052	0.874 ± 0.045	0.876 ± 0.044	0.853 ± 0.053	0.524 ± 0.166	0.213 ± 0.186	−0.637 ± 0.105
	BMH	0.853 ± 0.051	0.856 ± 0.050	0.862 ± 0.048	0.870 ± 0.046	0.846 ± 0.055	0.542 ± 0.162	0.180 ± 0.188	−0.618 ± 0.109
	CPH	0.814 ± 0.063	0.821 ± 0.062	0.786 ± 0.070	0.811 ± 0.063	0.825 ± 0.062	0.523 ± 0.167	0.025 ± 0.195	−0.533 ± 0.126
	BMH/BHP	0.633 ± 0.123	0.604 ± 0.133	0.575 ± 0.141	0.653 ± 0.121	0.668 ± 0.120	0.687 ± 0.139	0.038 ± 0.206	−0.541 ± 0.141
	BMH/CPH	0.296 ± 0.184	0.275 ± 0.186	0.408 ± 0.167	0.372 ± 0.172	0.194 ± 0.193	−0.009 ± 0.229	0.663 ± 0.140	−0.442 ± 0.161
	E	−0.762 ± 0.079	−0.712 ± 0.095	−0.849 ± 0.054	−0.858 ± 0.051	−0.733 ± 0.088	−0.601 ± 0.151	−0.492 ± 0.152	0.912 ± 0.032
	**Phenotypic**	**W**	**SL**	**BHP**	**BMH**	**CPH**	**BMH/BHP**	**BMH/CPH**	**E**
400 d	W	0.786 ± 0.018	0.765 ± 0.019	0.778 ± 0.021	0.783 ± 0.019	0.740 ± 0.021	0.337 ± 0.031	0.015 ± 0.044	−0.474 ± 0.046
	SL	0.791 ± 0.018	0.790 ± 0.017	0.781 ± 0.020	0.787 ± 0.019	0.746 ± 0.020	0.358 ± 0.028	0.013 ± 0.039	−0.430 ± 0.046
	BHP	0.798 ± 0.017	0.777 ± 0.019	0.809 ± 0.017	0.809 ± 0.016	0.752 ± 0.020	0.328 ± 0.032	0.059 ± 0.044	−0.536 ± 0.041
	BMH	0.798 ± 0.018	0.796 ± 0.017	0.803 ± 0.018	0.810 ± 0.017	0.751 ± 0.020	0.354 ± 0.031	0.056 ± 0.044	−0.533 ± 0.042
	CPH	0.761 ± 0.020	0.765 ± 0.020	0.748 ± 0.022	0.764 ± 0.020	0.739 ± 0.021	0.336 ± 0.031	−0.036 ± 0.042	−0.464 ± 0.045
	BMH/BHP	0.478 ± 0.030	0.474 ± 0.027	0.313 ± 0.031	0.496 ± 0.029	0.458 ± 0.030	0.385 ± 0.026	0.032 ± 0.035	−0.362 ± 0.038
	BMH/CPH	0.081 ± 0.043	0.072 ± 0.041	0.130 ± 0.044	0.118 ± 0.043	−0.001 ± 0.042	0.005 ± 0.031	0.306 ± 0.031	−0.197 ± 0.045
	E	−0.615 ± 0.035	−0.521 ± 0.037	−0.672 ± 0.029	0.001 ± 0.028	−0.574 ± 0.035	−0.274 ± 0.036	−0.175 ± 0.047	0.797 ± 0.021

## Data Availability

The dataset supporting this article has been uploaded as part of the electronic Supplementary Materials.

## Supplementary Materials

The following are available online at https://www.mdpi.com/article/10.3390/ani11051206/s1, Figure S1: Phenotypic data for growth traits (weight and SL), heights (BHP, BMH and CPH), height ratios (BMH/BHP and BMH/CPH) and ellipticity at 400 (A) and 800 d (B). Data are shown by EB and gender. The number (*n*) of soles evaluated in each batch is indicated below weight 400 d. The statistical significance of the three fixed factors (gender (G), evaluation batch (EB) and amoebic disease (AD)) is boxed in each trait. The letters denote significant differences between EB. The asterisks indicate significant differences between gender when interaction G × ΕB is significant; Figure S2: Regressions of log standard length (Log SL) on log weight (Log W) for males and females at 400 and 800 d. The slope and the determination coefficient are indicated; Figure S3: Regressions of BHP on BMH (A,B) and CPH on BMH (C,D) for males and females at 400 (A,C) and 800 d (B/D). The regression equations and determination coefficients are indicated; Figure S4: Estimated marginal means for BMH/BHP on BMH/CPH ratios as determined by repeated-measures ANOVA at 400 and 800 d for the (A) gender (male; female), (B) EB (1–7) and (C) AD (infected vs. non-infected). The asterisks on the horizontal or vertical arrows denote if within- or between-subject effects were significant; Figure S5: Comparison of sole shape with a low (A) vs. high (B) BMH/CPH ratio. The weight (W) and Ellipticity (E) (top) and the BMH/CPH (left down) and BMH/BHP (right down) ratios are indicated. The theoretical ellipse that fits the mouth tip and the middle of peduncle with the BMH is shown in red dashed line.
