# Supplementary figures and images for: Genetic Estimates for Growth and Shape-Related Traits in the Flatfish Senegalese Sole
Source: Animals (Basel). 2021 Apr 22;11(5):1206. doi: 10.3390/ani11051206 (PMC8146546; doi:10.3390/ani11051206)

A) 400 d

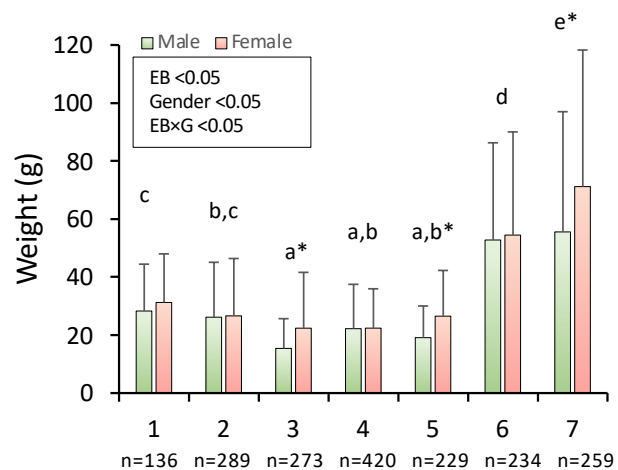

B) 800 d

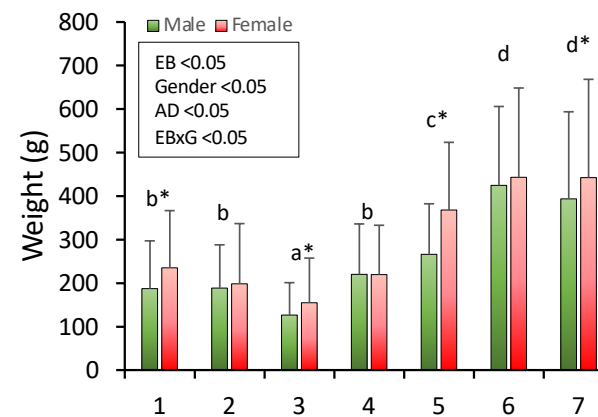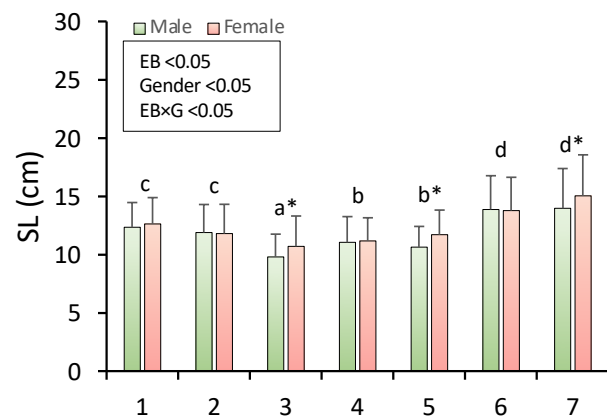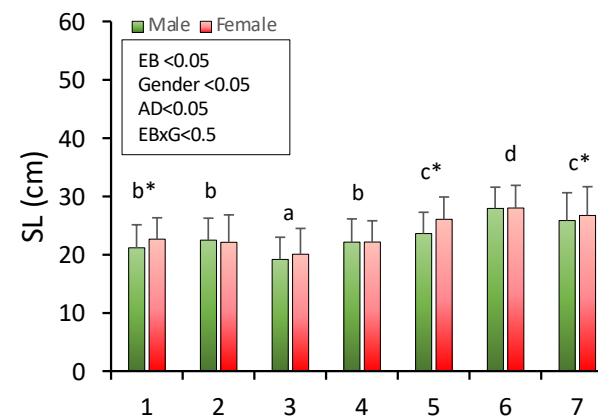

A) 400 d

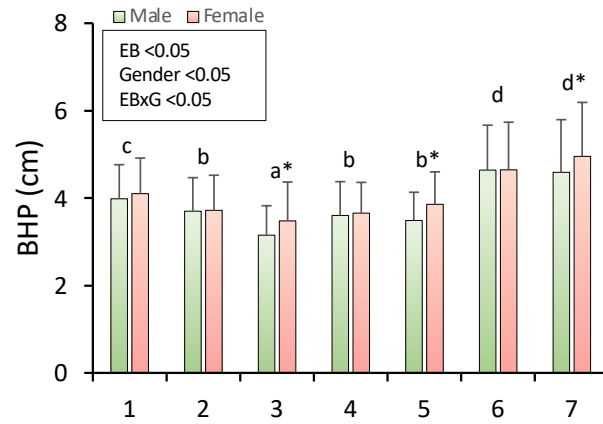

B) 800 d

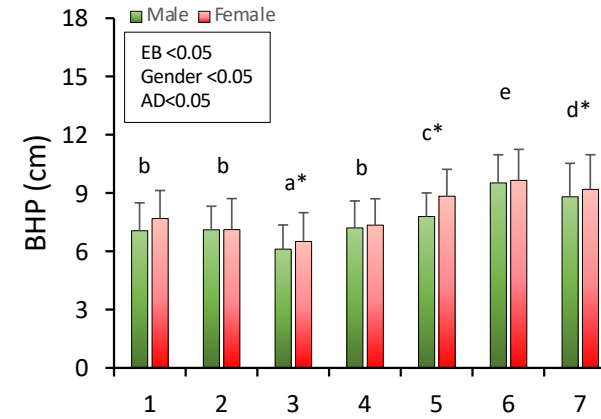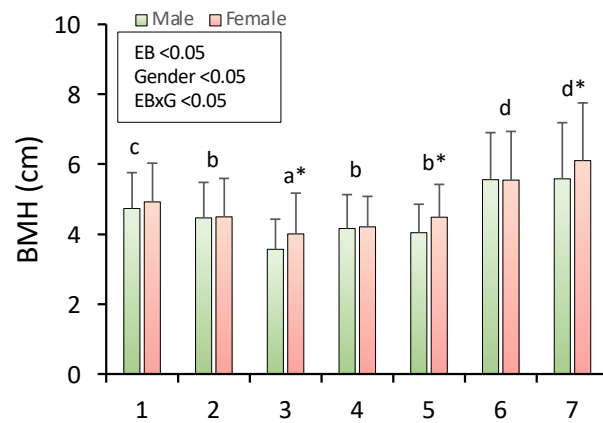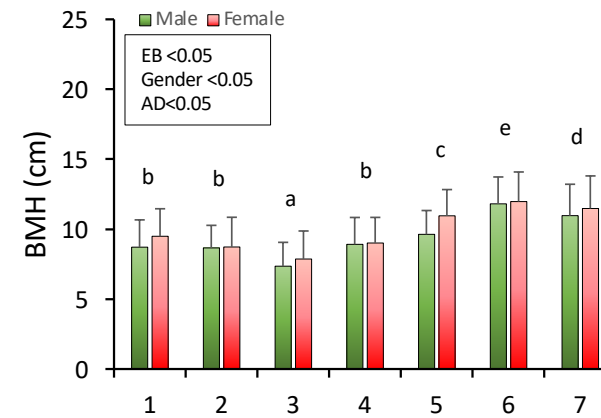

A) 400 d

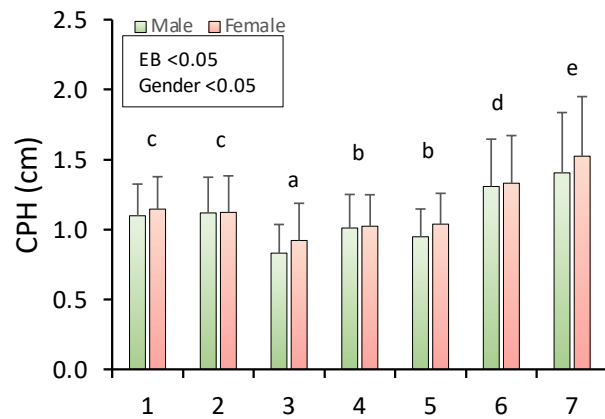

B) 800 d

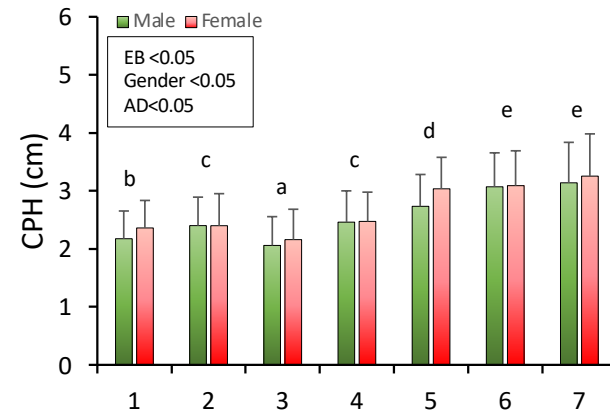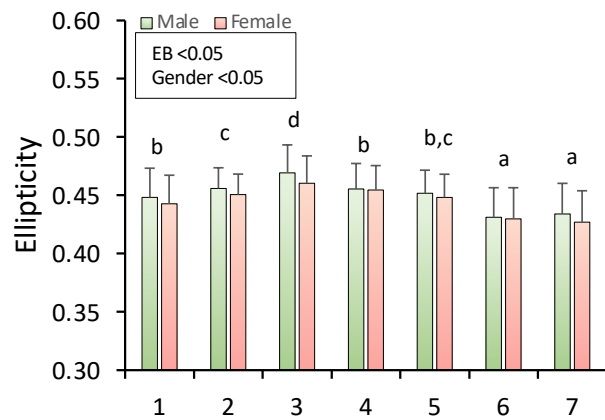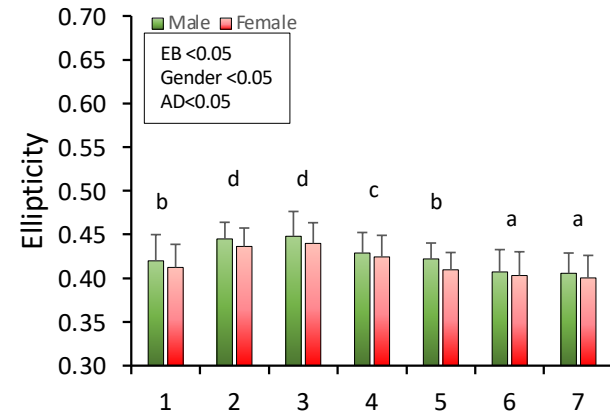

A) 400 d

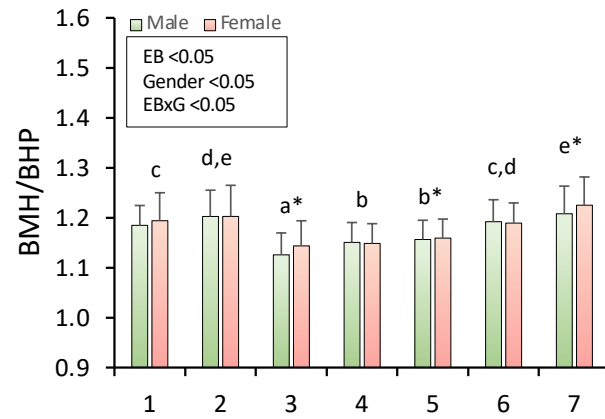

B) 800 d

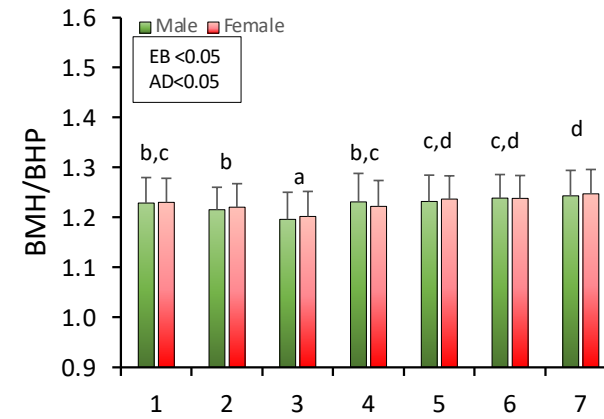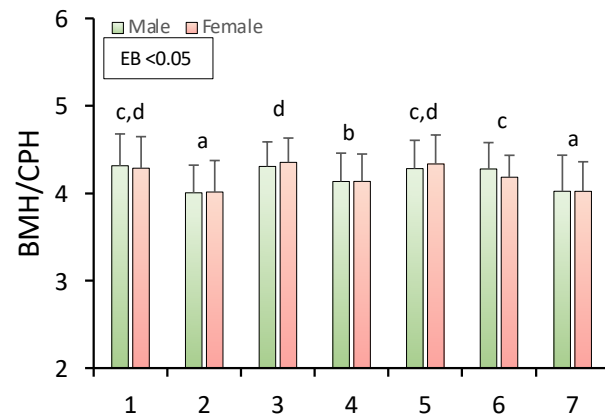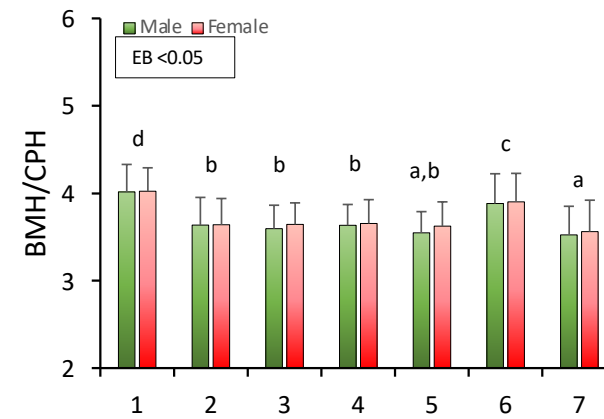

Supplement: Supplementary file 1 [file animals-11-01206-s001.zip › Figure S1.pdf]

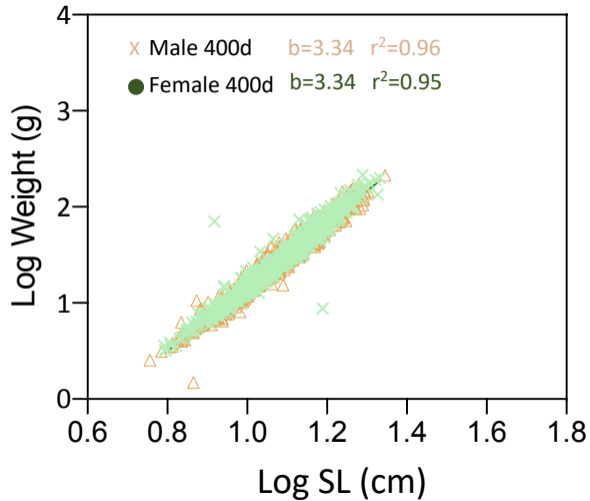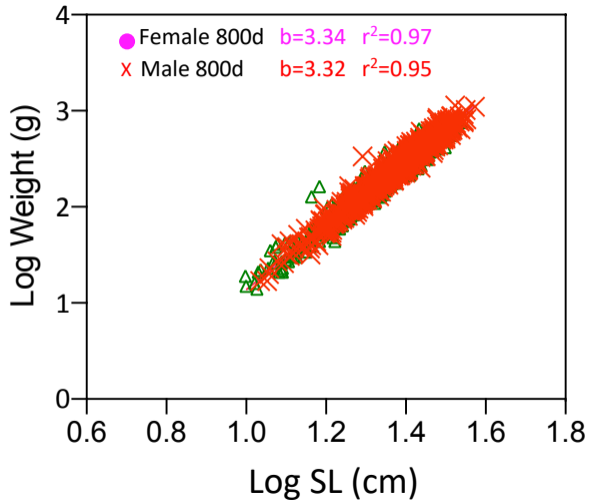

Supplement: Supplementary file 1 [file animals-11-01206-s001.zip › Figure S2.pdf]

A)

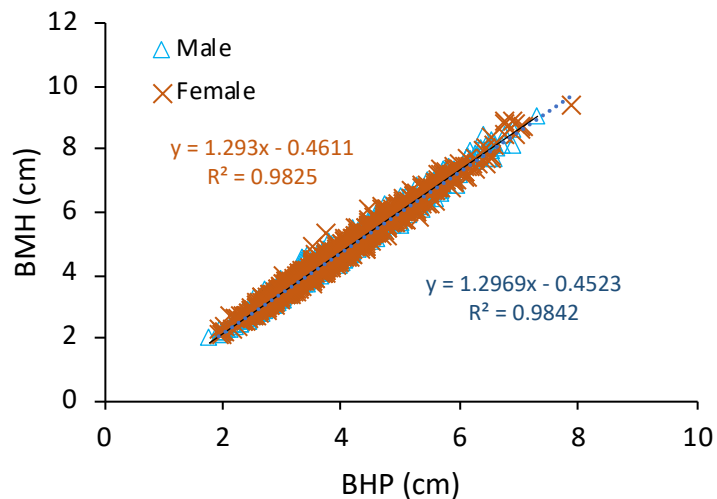

B)

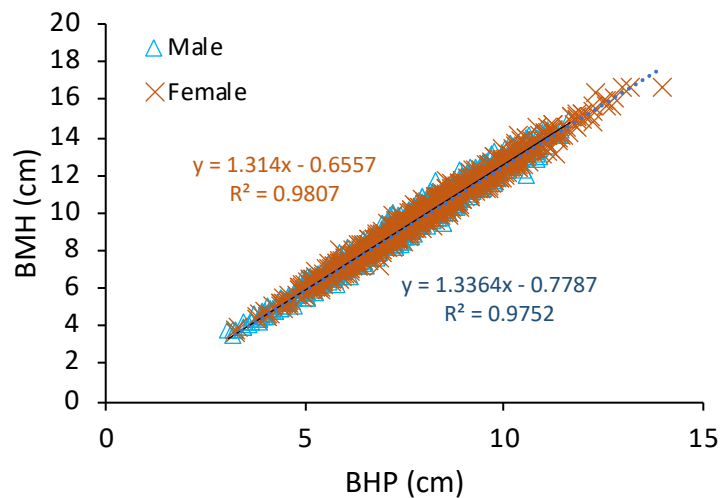

C)

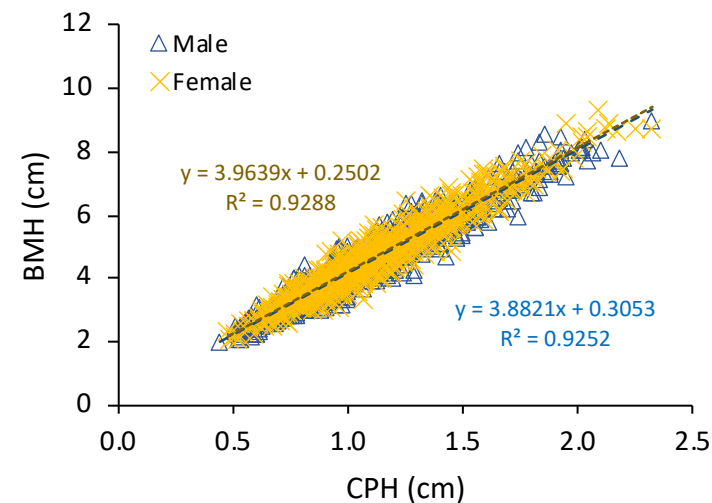

D)

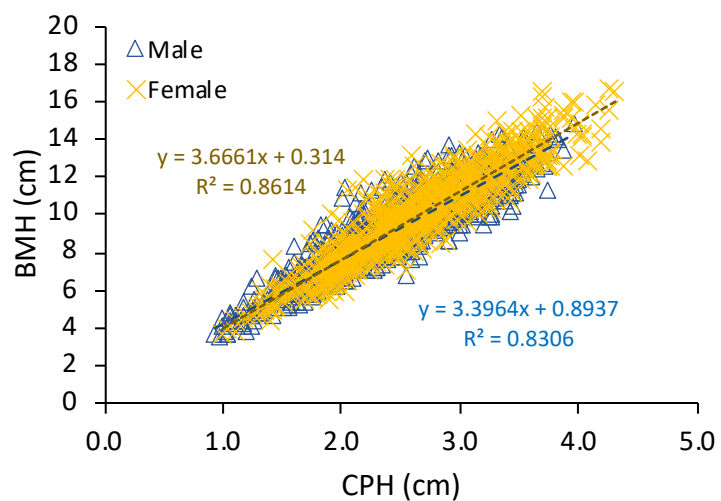

Supplement: Supplementary file 1 [file animals-11-01206-s001.zip › Figure S3.pdf]

A)

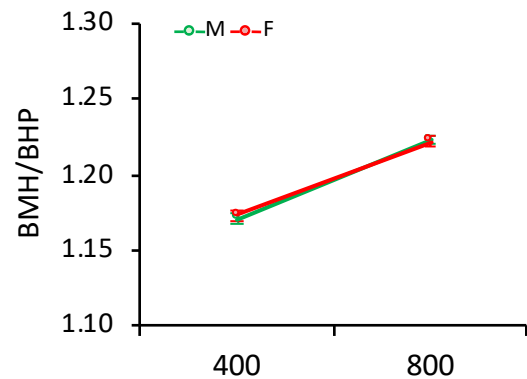

B)

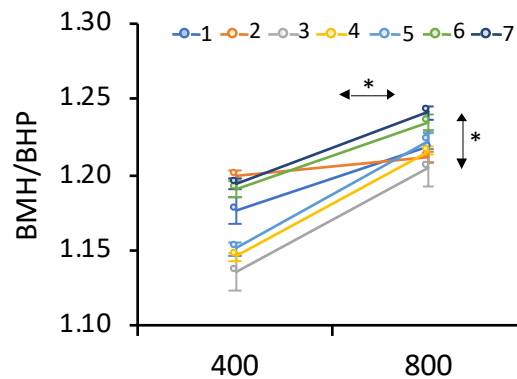

C)

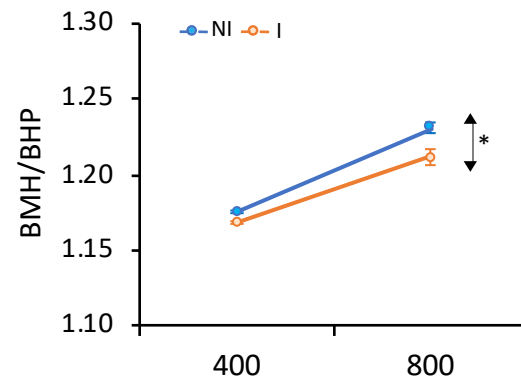

A)

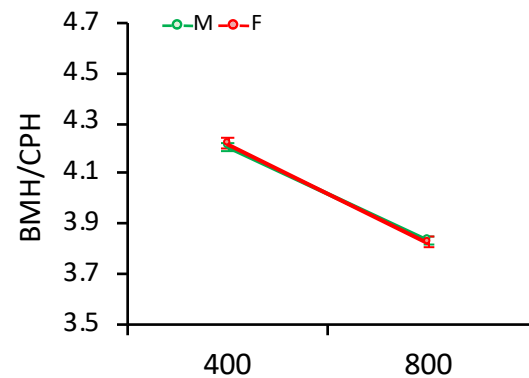

B)

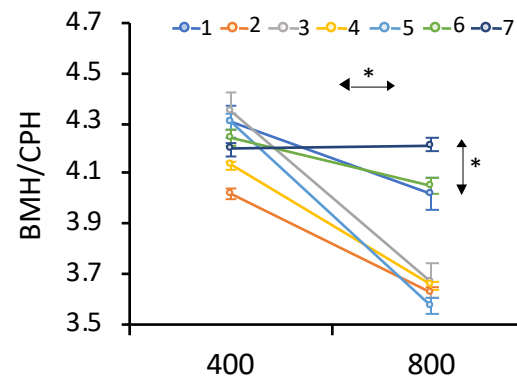

C)

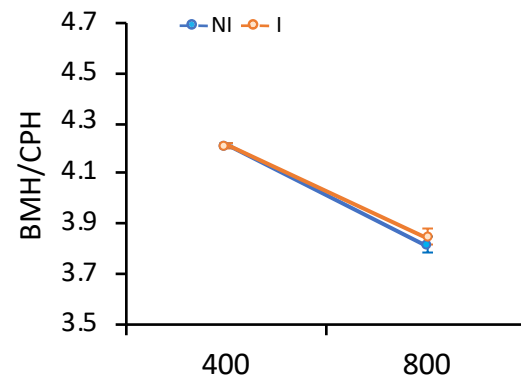

Supplement: Supplementary file 1 [file animals-11-01206-s001.zip › Figure S4.pdf]

A)

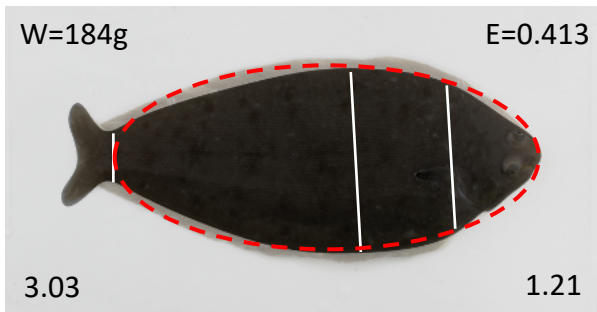

B)

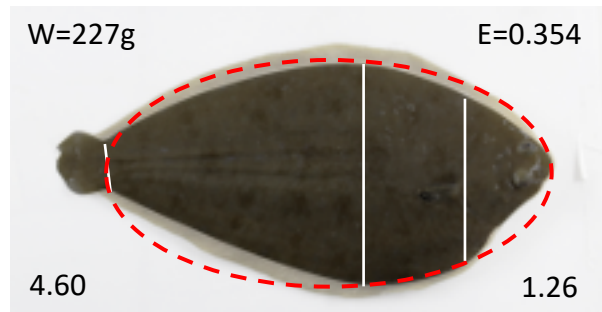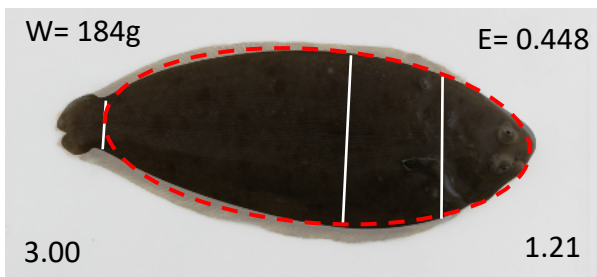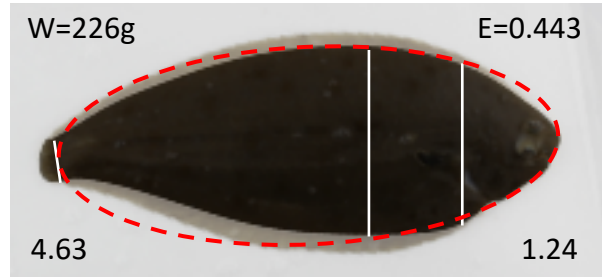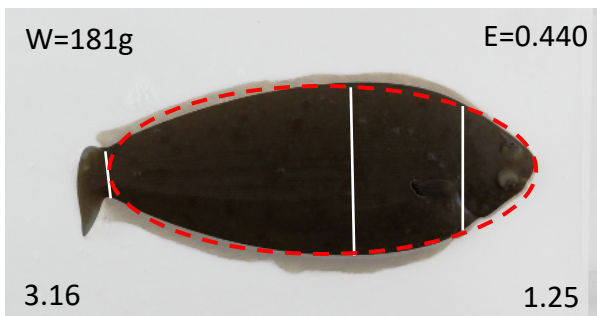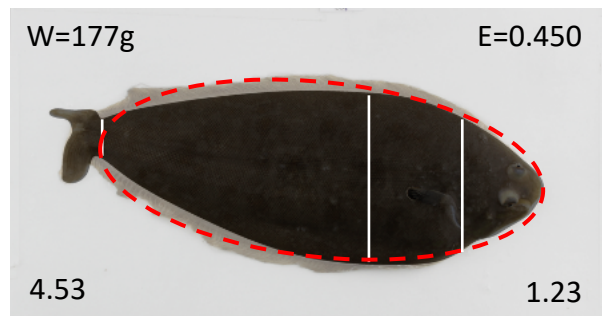

Supplement: Supplementary file 1 [file animals-11-01206-s001.zip › Figure S5.pdf]
